# Lifestyle and reproductive risk factors associated with anal cancer in women aged over 50 years

**DOI:** 10.1038/bjc.2015.89

**Published:** 2015-03-12

**Authors:** K Coffey, V Beral, J Green, G Reeves, I Barnes

**Affiliations:** 1 Cancer Epidemiology Unit, University of Oxford, Richard Doll Building, Roosevelt Drive, Oxford OX3 7LF, UK

**Keywords:** anal cancer, human papillomavirus, Million Women Study, epidemiology

## Abstract

**Background:**

Anal cancer incidence increases with age and is higher in women than men. Risk factors in this group other than high-risk human papillomavirus infection are unclear.

**Methods:**

In all, 1.3 million women were recruited in 1996–2001 and followed for incident anal cancer. Cox regression models were used to calculate relative risks (RRs) for anal cancer by various potential risk factors.

**Results:**

Five hundred and seventeen incident anal cancers were registered over 13 years of follow-up. The largest RR was associated with a history of cervical intraepithelial neoplasia grade 3 (CIN 3; RR=4.03, 95% CI 2.59–6.28). Other factors associated with significantly increased risks in multivariate analyses were: ever smoking (RR=1.49, 1.24–1.80); previous use of oral contraceptives (RR=1.51, 1.24–1.83); nulliparity (RR=1.61, 1.24–2.07); tubal ligation (RR=1.39, 1.13–1.70) and not living with a partner (RR=1.82, 1.40–2.38). The association with smoking was significantly greater for squamous cell carcinoma than adenocarcinoma of the anus (RR 1.66 *vs* 0.89, *P* for heterogeneity=0.04).

**Conclusions:**

History of CIN 3, smoking, past oral contraceptive use, nulliparity, tubal ligation and not living with a partner are risk factors for anal cancer in women. There was a significant increase in risk associated with smoking for squamous cell anal cancers but not adenocarcinomas.

In the past 20 years there has been more than a doubling in incidence of female squamous cell anal cancer registrations in the UK, the rise being most marked in middle-aged and older women (Wilkinson *et al*, 2014). Incidence rates increase rapidly with age, and women in England have higher rates of anal cancer than men (see Figure 1, based on data from the Office of National Statistics, 2014). There are very few cases of anal cancer before age 40; in England in 2012 64% (665) of the 1043 registered anal cancer cases occurred in women and 88% (584) of the female cases were in women aged over 50 years.

High-risk human papillomavirus (HrHPV) infections, which cause the majority of cases of cervical cancer (Jacobs *et al*, 1999), are also responsible for between 80 and 90% of anal cancers (International Agency for Research on Cancer, 2005; Steinau *et al*, 2013). Similarly, HPV 16 and 18 appear to be the commonest HPV types associated with anal tumours (Steinau *et al*, 2013). Despite the evidence of a strong association between high-risk strains of the HPV virus and several cancers, including anal cancer, HrHPV oncogenesis is puzzling; the majority of sexually active women are exposed to HPV infections during their lifetimes (Baseman and Koutsky, 2005), but the anogenital cancers they cause remain relatively rare. Identification of other risk factors that have a role in HPV-related anogenital carcinogenesis is clearly important if we wish to identify individuals with an increased risk of developing an anal malignancy.

Several high-risk populations for anal cancer are already known, including human immunodeficiency virus (HIV)-positive individuals (Silverberg *et al*, 2012), organ transplant recipients (Madeleine *et al*, 2013), men who have sex with men (MSM) (Machalek *et al*, 2012), and women with a history of preinvasive or invasive cervical or vulval lesions (Ouhoummane *et al*, 2013). Most published research has focused on these groups, particularly HIV-positive MSM (Stanley *et al*, 2012). The literature on the role of anal HPV infection in non-HIV infected individuals has also mostly examined risk in men, with twice as many studies involving males than females (Giuliano *et al*, 2014). Although older women account for a large proportion of those developing anal cancer, they have not been the focus of previous large-scale epidemiological research.

Other than HrHPV infection, risk factors for anal cancer in women, particularly older women who have the highest incidence of anal cancer, are unclear.

Our aim is to examine reproductive, lifestyle, hormonal, and other risk factors for incident anal cancers in women aged over 50 years in a large cohort of UK women.

## Materials And Methods

The Million Women Study is a population-based prospective study, which recruited women via the National Health Service Breast Screening Programme from 1996 to 2001. The study was established to examine the association between menopausal hormone therapy and breast cancer, and to investigate associations between lifestyle, reproductive and hormonal risk factors, and other health outcomes (The Million Women Study Collaborative Group, 1999).

Women were invited to participate in the study via a postal questionnaire, which was distributed with their usual breast-screening invitation. Full details of the study design and methods are described in detail elsewhere (Beral and The Million Women Study Collaborative Group, 2003). About 1.3 million women returned a completed questionnaire. Participants gave written consent for use of their questionnaire data for research, and for ongoing linkage to nationally held registry and health data. The Study has Multi-Centre Research Ethics Committee approval (97/01).

The main analysis excluded participants if they had breast or any other invasive cancer registered prior to recruitment, with the exception of non-melanoma skin cancer (International Classification of Diseases, 10th revision; ICD-10 C44). Self-reported data from the recruitment and subsequent resurvey questionnaire were used to define most exposures; exposures were not updated. Deprivation index was calculated using post-code and 1991 census data (Townsend, 1987).

Million Women Study participants are flagged on the UK National Health Service Central Registers, which provide the study team with regularly updated information on incident cancers and deaths. Cases of incident anal cancer are those coded as C21 ‘malignant neoplasm of anus and anal canal' according to the (ICD-10) (World Health Organization, 2011). Cervical intraepithelial neoplasia grade 3 (CIN 3) is routinely captured by the UK cancer registry as an *in situ* cancer: CIN 3 registrations (ICD-10 D06) that occurred prior to recruitment were also identified from the cancer registry data.

Risk factors with sufficient numbers of cases in exposed and unexposed women were also examined separately for the two most common histological subtypes of anal cancer: squamous cell carcinoma and adenocarcinoma. These were identified using ICDO-3 (ICD for Oncology version 3) morphology codes. Squamous tumours included the following ICDO-3 codes: M80810/3, M8070/3, M8071/3, M8072/3, M8083/3, M8094/3, M8123/3, and M8124/3. Adenocarcinomas included codes M8140/3, M8210/3, M8263/3, M8480/3, and M8481/3.

### Statistical analyses

Cox proportional hazards models were used to examine the relationship between exposures and subsequent risk of anal cancer. Hazard ratios, described here as relative risks (RRs), were estimated with attained age as the underlying time variable. Participants entered the analysis from the date they reported the relevant exposure (which was the date they filled in the questionnaire, either at recruitment or subsequent resurvey). The small number of women who were under the age of 50 at recruitment (3%) entered the analysis at age 50. Women were followed to the earliest of date of first cancer registration, death, emigration, or 31 December 2012, the date of the most recent ONS follow-up.

Analyses were stratified by region of residence (10 geographical areas which correspond to the cancer registry regions: Oxford, East Anglia, South West, Thames, West Midlands, Northern & Yorkshire, Trent, North West (Mersey), North West (Manchester/Lancashire), Scotland) and adjusted where appropriate for previous registration of CIN 3 (yes/no); smoking (never *vs* ever); oral contraceptive pill use (never *vs* ever); parity (parous *vs* nulliparous); tubal ligation (no/yes); deprivation (quintile of Townsend Deprivation Index); age at menarche (⩽12, 13, 14+); alcohol use (0–2 units per week, 3–7 units per week, 8+ units per week); and body mass index (<25, 25–30, 30+). Where smoking and oral contraceptive pill use were examined as exposures, responses were categorised by amount smoked: smoking (never, past, current <15 cigarettes per day, current >15 cigarettes per day); and duration of use oral contraceptive pill use (none, <5 years, 5+ years).

Age at menopause (50+, 45–49, <45) and hormone therapy use (never/ever) were examined as exposures in postmenopausal women only; the association with age at menopause was further restricted to naturally postmenopausal women or those reporting a bilateral oophorectomy, who had never used hormone therapy. Apart from CIN 3 registration and deprivation quintile, these exposures were all based on self-reported questionnaire data collected from participants at baseline.

We also examined the impact of being married or living with a partner (yes/no), and of self-reported attendance for cervical cancer screening (ever/never) in fully adjusted models. These exposures were asked about on the first resurvey, which was sent out on average 3 years after recruitment.

The small number of women with missing data (fewer than 2% for each variable) were included in a separate category for each variable of interest and included in the analysis but these data are not shown. Relative risks are reported with 95% confidence intervals. Analyses were performed in Stata 13 (StataCorp, 2013).

We used a competing hazards model to examine the interaction between anal cancer histological subtype and past CIN 3, smoking, and oral contraceptive use.

## Results

In all, 1.3 million women were followed for 16.9 million person-years, giving an average of 13 years of follow-up per woman. During this time, 517 were registered with incident anal cancer. Table 1 shows participant characteristics at baseline. Mean age at recruitment was 56.1 (s.d. 4.9); 46% reported ever having smoked, with 19% currently smoking at recruitment; 59% reported ever using oral contraceptives, and 33% reported >5 years of use. Most women had given birth at least once, with 11% reporting being nulliparous.

**Figure 2:**
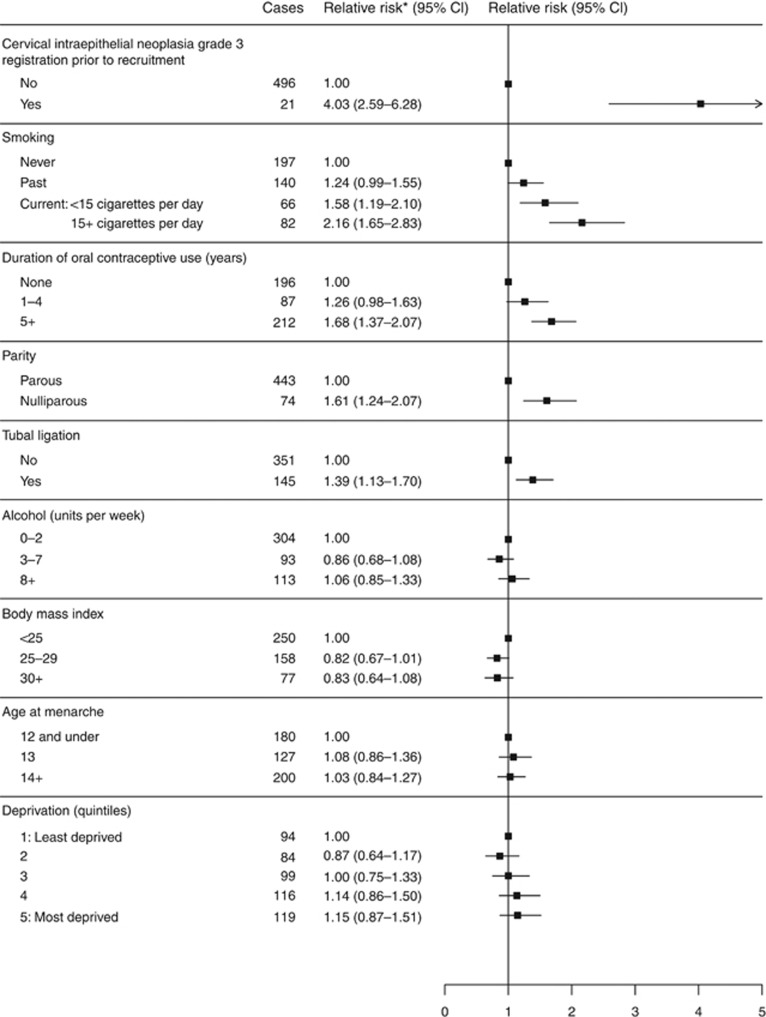
**Association between anal cancer and various lifestyle and other factors.** *Estimates of relative risk are adjusted by age, region of residence, and all other factors shown above, where appropriate.

About 1% (12 531) of the women in the cohort had a registration of CIN 3 prior to recruitment, and this was the strongest predictor of risk of anal cancer after the age of 50 (RR=4.03, 95% CI 2.59–6.28, *P*<0.0001), after adjustment for smoking, alcohol use, BMI, age at menarche, ever use of the oral contraceptive pill, parity, tubal ligation, and deprivation (Figure 2).

Ever having smoked was associated with a 50% increase in risk of subsequent anal cancer, with a RR of 1.49 (95% CI 1.24–1.80; *P*<0.0001) compared with never smokers. Current smokers who reported smoking >15 cigarettes per day at recruitment had more than a doubling of risk (RR=2.16, 1.65–2.83; *P*<0.0001) (Figure 2). No significant increase in risk was seen with alcohol use, adiposity, age at menarche, or deprivation.

Past use of the oral contraceptive pill was significantly associated with anal cancer risk (RR=1.51, 1.24–1.83; *P*<0.0001) for ever *vs* never use. When stratified by years of use, women who reported 5 or more years of oral contraceptive use had a significantly increased risk of anal cancer, RR=1.68 (95% CI 1.37–2.07; *P*<0.0001) (see Figure 2).

Overall, nulliparous women had a 60% greater risk of anal cancer when compared with parous women, with a RR of 1.61 (95% CI 1.24–2.07; *P*<0.0001). Women who reported ever having given birth had a lower risk of developing anal cancer, irrespective of the number of children they had, with a RR of 0.58 (95% CI 0.44–0.75; *P*<0.0001) for those reporting 1–2 children, and a RR of 0.69 (95% CI 0.52–0.91; *P*<0.0001) for women with 3+ children, compared with the nulliparous group.

Female sterilisation was also associated with an increased risk of anal cancer; women who reported having had a bilateral tubal ligation were significantly more likely to develop an anal cancer, with a RR of 1.39 (95% CI 1.13–1.70; *P*<0.0001). Neither age at menopause nor use of hormone therapy was significantly associated with anal cancer risk (Table 2).

At resurvey, on average 3 years after recruitment, women were asked whether they were currently married or living with a partner. About a fifth (19%) reported not living with a husband or partner, and their risk of anal cancer was increased (RR of 1.82, 1.40–2.38; *P*<0.0001) (Table 3). Only 4% reported never having had a previous cervical screening (smear) test. The risk of anal cancer was not significantly different in women who had and had not been screened (Table 3).

We also compared associations for squamous cell cancers (SCC) *vs* adenocarcinomas of the anus (Table 4). The majority of the anal cancers registered in the cohort (*n*=425, 82%) were SCCs. Glandular tumours were less common, with 62 adenocarcinomas (12%) registered. The remaining 27 (5%) were rarer histological subtypes, including anal melanoma; this group had insufficient numbers to examine separately.

The RR of adenocarcinoma of the anus in women who reported any lifetime smoking was 0.89 (95% CI 0.51–1.54), which was significantly different (*P* for heterogeneity=0.04) from the risk in women with squamous cell tumours (RR 1.66, 95% CI 1.35–2.05). In contrast, the association between past CIN 3 and oral contraceptive use were similar in the two subtypes. We were not able to examine all factors by histological subtype; the other exposures that showed a positive association with anal cancer (tubal ligation, parity, and not living with a husband or partner) had an insufficient number of cases of adenocarcinoma to permit valid subgroup analysis.

## Discussion

We examined lifestyle, reproductive, and other risk factors for incident anal cancer in a large prospective study of UK women aged over 50 years. Factors significantly associated with an increased risk of anal cancer were: prior high grade cervical disease (CIN 3); ever having smoked, with a doubling of risk in current smokers of >15 cigarettes a day; ever having used the oral contraceptive pill; nulliparity; a history of tubal ligation; and not currently living with a husband or partner. Among smokers, the risk of squamous cell anal carcinoma differed significantly from the risk of adenocarcinoma, with an elevated risk of squamous cell tumours but not of adenocarcinoma.

The strongest association with risk of anal cancer in our study was in the 1% of women who had a registration of CIN 3 before they were recruited. Women with HrHPV-associated neoplasia in one part of the anogenital tract are known to be more likely to have it elsewhere (Crawford *et al*, 2011). After cervical cancer, anal cancer has the closest association with HrHPV infection (Grulich *et al*, 2012), with an estimated 80–90% of anal cancers caused by oncogenic papillomaviruses (International Agency for Research on Cancer, 2005; Steinau *et al*, 2013; Stewart and Wild, 2014).

Anal cancer, and other HPV-related anogenital cancers are increased in women with a history of anogenital warts (Blomberg *et al*, 2012), cervical dysplasia (Kalliala *et al*, 2005; Edgren and Sparén, 2007), and previous anogenital malignancy at another site (Frisch *et al*, 1994; Jiménez *et al*, 2009; Saleem *et al*, 2011). In the United Kingdom, CIN 3 but not other grades of cervical dysplasia are registered, so we were not able to examine the risk associated with any other grade of CIN. There are no data on the completeness of CIN 3 registration. Cases are notified to the cancer registries by pathology laboratories, and it is possible that some cases may have been missed.

Though smoking has been found to be a risk factor for anal cancer in some other studies (Daling *et al* 1987, 1992, 2004; Frisch *et al*, 1999), not all have reported such an association (Ouhoummane *et al*, 2013). A recent Brazilian retrospective study reported a doubling of anal cancer risk in women associated with smoking, similar to our findings (Moura *et al*, 2014). Another retrospective study from Denmark and Sweden (Frisch *et al*, 1999) with 417 anal cancer patients (324 women and 93 men) also found about a doubling of risk of anal cancer in female smokers. Laboratory studies have shown increased concentrations of nicotine and other metabolites in the cervical mucus of smokers (Sasson *et al*, 1985; Hellberg *et al*, 1988; Prokopczyk *et al*, 1997), suggesting a potential biological pathway for the increase in risk seen in cervical cancer, which may also apply to anal epithelium. This could be through direct carcinogenesis with constituents of tobacco acting as carcinogens; indirectly through suppression of the immune response to HrHPV; or through some other mechanism.

We also found that the risk associated with smoking varies significantly by tumour histology, with an increase in anal SCCs, but not in adenocarcinomas. Similar heterogeneity has previously been reported for cervical cancer—smoking was significantly related to cervical SCCs but not adenocarcinomas (International Collaboration of Epidemiological Studies of Cervical Cancer, 2006). The heterogeneity in risk by histological subtype observed here for anal cancers, and previously seen in cervical cancers, suggests that anogenital cancers arising in squamous and glandular issues may have different biological pathways. It has been suggested that the majority of cases of anal adenocarcinoma actually represent downward spread from rectal tumours (International Agency for Research on Cancer, 2009). In this study, 8% of the adenocarcinoma cases (5/62) had ICDO-3 morphology codes that are usually associated with rectal (ICD-10 C18-20) and not anal cancers (ICD-10 C21).

Past use of oral contraceptives was a clear predictor of risk of subsequent anal cancer in our study, particularly for women with longer-term use of 5 or more years. This is a little-investigated association; we found only one previous study of anal cancer risk that mentioned oral contraceptive use, and no significant association was found (Frisch *et al*, 1999). Oral contraceptives have been shown to be associated with an increased risk of cervical cancer during use and for a period of time after cessation (International Collaboration of Epidemiological Studies of Cervical Cancer, 2007). It has been suggested that sex hormones may stimulate HrHPV gene expression or exert an effect on the immune microenvironment in the cervical epithelium (Delvenne *et al*, 2007), and it is possible that a similar effect could occur in anal epithelium.

The increased risk of anal cancer in women who reported a history of tubal ligation has not, to our knowledge, previously been reported. Tubal ligation is highly correlated with parity; over 97% of the women in our cohort who reported being sterilised had given birth to at least one child. Sterilised women had more children, with 49% reporting three or more children, compared with 33% of women in the cohort overall. Given that in our analysis the risk of anal cancer was decreased in parous women, the relationship between tubal ligation and increased risk was unexpected. Adjusting for parity, age at first birth, or number of children did not affect the association.

HPV infections are sexually transmitted. The risk of anal cancer and its precursor lesions have been shown to be closely related to sexual behaviour in both women and men, including number of partners and sexual practices (Holly *et al*, 2001; Tseng *et al* 2003; Grulich *et al*, 2012). A limitation of our study is that we did not collect information on sexual behaviour from our participants. We examined current marital status and co-habitation, and found that women who reported not currently being married or living with a partner had an increased risk of subsequent anal cancer.

Given that HPV is sexually transmitted, this might seem counter-intuitive; however, most women aged over 50 years who are not married or living with a partner are widowed, separated, or divorced (Floud *et al*, 2014). Being separated or divorced has been reported to be associated with an increased risk of anal cancer in both men and women; Peters and Mack (1983) found a doubling of risk for squamous cell anal cancers in separated or divorced women. Cervical cancer is also more common in separated and divorced than in currently married women (Beral, 1974), and it seems probable that differences in past sexual behaviour account for the findings related to marital status at older ages.

In a large cohort of over a million women with prospectively collected information on exposures, with an average 13 years of follow-up, we found that prior CIN 3, smoking, past use of the oral contraceptive pill, nulliparity, tubal ligation, and not currently living with a husband or partner were risk factors for incident anal cancer in women over 50 years of age. Anal cancer has the closest relationship to HrHPV after cervical cancer (Grulich *et al*, 2012), and it is possible that the risk factors we have identified are also associated with the risk of HPV acquisition, and/or a decreased clearance of existing infection, leading to the increased risk of anal cancer seen in association with certain exposures, or that some of these exposures themselves increase risk of anal cancer.

When we looked separately at the two main histological subtypes of anal cancer, we found a significant difference in risk associated with smoking for tumours with squamous cell morphology compared with adenocarcinomas. To our knowledge, this has not previously been reported in anal cancer; however, similar heterogeneity between squamous and adenocarcinomas has been reported for other cancers, including cancer of the cervix (International Collaboration of Epidemiological Studies of Cervical Cancer, 2006), and lung, where squamous cell tumours have been found to be more strongly associated with smoking (Houston *et al*, 2014).

Anal cancer, like the other anogenital cancers, has a strong relationship with high-risk strains of HPV. HrHPV infections are extremely common, but the cancers they cause are rare. We have identified several additional risk factors with strong associations to anal cancer risk in older women, particularly smoking and past oral contraceptive use.

## Figures and Tables

**Figure 1:**
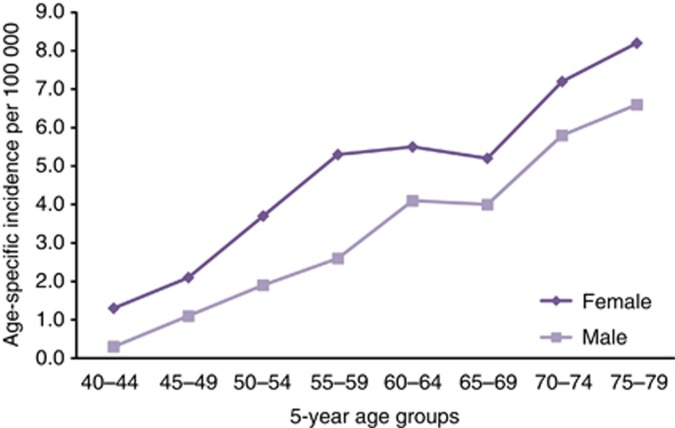
Age-specific rates of anal cancer per 100 000 population, England 2012 (ONS, 2014).

**Table 1:** Participant characteristics at recruitment

**Number of women**	**1 300 101**	
**Age in years—mean (s.d.)**	**56.1**	**4.9**
	***N***	**%**
Body mass index >30	220 986	17.0
Ever used oral contraceptives	761 728	58.6
Ever smokers	596 284	45.9
Parous	1 157 243	89.0
Three or more children	427 331	32.9
History of tubal ligation	289 634	22.3
Hysterectomy	316 944	24.4
Postmenopausal, never HT user	424 872	32.7
Ever used hormone therapy	644 831	49.6
Most deprived quintile	255 619	19.7
Prior cervical intraepithelial neoplasia grade 3 registration	12 531	1.0

Abbreviation: HT=hormone therapy.

**Table 2:** Association between anal cancer and risk factors associated with menopause

**Exposure**	**Cases/population at risk**	**RR**	**95% confidence interval**
**Age at menopause in never users of hormone therapy in women with natural menopause or bilateral oophorectomy**			
50+	80/237 157	1.00	—
45–49	52/126 008	1.18	0.83–1.68
<45	18/49 489	0.95	0.45–1.59
**Use of hormone therapy at baseline, all postmenopausal participants**			
Never	189/497 518	1.00	—
Ever	276/625 025	1.19	0.98–1.44

Abbreviation: RR=relative risk. Adjusted for smoking, alcohol use, BMI, OC use, age at menarche, parity, tubal ligation, previous CIN 3, deprivation and stratified by region.

**Table 3:** Association between living with a partner and participation in cervical screening on incidence of anal cancer in respondents to resurvey 3 years after recruitment

**Exposure**	**Cases/population at risk**	**RR**	**95% confidence interval**
**Married or living with a partner**			
Yes	178/638 188	1.00	—
No	88/153 782	1.82	1.40–2.38
**At least one prior cervical smear test**			
Yes	177/558 237	1.00	—
No	7/23 997	0.84	0.39–1.81

Abbreviation: RR=relative risk. Adjusted for smoking, alcohol use, BMI, OC use, age at menarche, parity, tubal ligation, previous CIN 3, deprivation and stratified by region.

**Table 4:** Associations for anal cancer by tumour histology

	**Squamous cell carcinoma**			**Adenocarcinoma**			
**Exposure**	**Cases/population at risk**	**RR**	**95% CI**	**Cases/population at risk**	**RR**	**95% CI**	**Heterogeneity by tumour type** ***P-*****value**
**Past CIN 3 registration**							
No	406/1 287 570	1.00	—	61/1 287 509	1.00	—	0.51
Yes	19/12 531	4.43	2.79–7.05	1/12 531	2.12	0.29–15.42	
**Ever smoked**							
Never	151/627 169	1.00	—	32/627 169	1.00	—	0.04
Ever	253/596 284	1.66	1.35–2.04	23/596 284	0.89	0.51–1.54	
**Oral contraceptive use**							
Never	138/523 851	1.00	—	24/523 851	1.00	—	0.74
Ever	282/761 728	1.52	1.23–1.89	38/761 728	1.68	0.98–2.88	

Abbreviations: CI=confidence interval; RR=relative risk. Adjusted for smoking, alcohol use, BMI, OC use, age at menarche, parity, tubal ligation, previous CIN 3, deprivation and stratified by region.
