# Lifestyle and reproductive risk factors associated with anal cancer in women aged over 50 years

**DOI:** 10.1038/bjc.2016.13

**Published:** 2016-05-26

**Authors:** K Coffey, V Beral, J Green, G Reeves, I Barnes

**Correction to**: *British Journal of Cancer* (2015) **112**, 1568–1574; doi:10.1038/bjc.2015.89

**Updated online 26 May 2016:** This article was originally published under a CC BY-NC-SA 4.0 license, but has now been made available under a CC BY 4.0 license. The PDF and HTML versions of the paper have been modified accordingly.

